# Sweet syndrome in a patient with Hidradenitis Suppurativa

**DOI:** 10.1002/ccr3.2724

**Published:** 2020-02-12

**Authors:** Lakshay Jain, Sreenath Meegada

**Affiliations:** ^1^ Internal‐Medicine UT Health East Texas/Christus Good Shepherd Medical Center Longview TX USA

**Keywords:** cellulitis, Hidradenitis suppurativa, Sweet syndrome

## Abstract

Neutrophilic Dermatoses should be considered in the differential diagnosis, if a patient with abrupt onset of painful erythematous plaques/nodules and elevated erythrocyte sedimentation rate is not responding to antibiotics.

## Graphical abstract

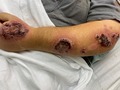

A 49‐year‐old man with history of Hidradenitis suppurativa presents to the emergency room with a 1‐week history of progressive worsening bilateral forearm rash. The lesions initially began as 1‐ to 2‐cm hypopigmented lesions over his left antecubital region and extended down to his left hand and ultimately to his right arm as well in a similar distribution. Review of systems was negative for fevers, trauma, or any recent travel. Physical examination showed exquisitely tender, edematous, and inflamed papules, plaques, and ulcerated areas with areas of central necrosis and crusting (Figures 1 and 2). Patient was initially treated with empiric antibiotics thinking it could be skin infection with no response. We later considered other differential diagnoses including pyoderma gangrenosum, neutrophilic eccrine hidradenitis, Behcet's syndrome, urticarial vasculitis, and Sweet syndrome. Skin biopsy showed pseudoepitheliomatous hyperplasia with intense inflammatory neutrophil infiltrates and reactive keratinocytes (Figure 3). The diagnosis of Sweet syndrome was made in this patient based on abrupt onset of painful erythematous plaques or nodules (major criteria), histopathological evidence of dense neutrophil infiltrate (major criteria), elevated erythrocyte sedimentation rate (ESR) of 130 mm/h (minor criteria), and excellent response to steroids (minor criteria).^1^ Patient was started on prednisone 60 mg daily with significant improvement in 48 hours and was discharged home on slow steroid taper for two more months.^2^

**Figure 1:**
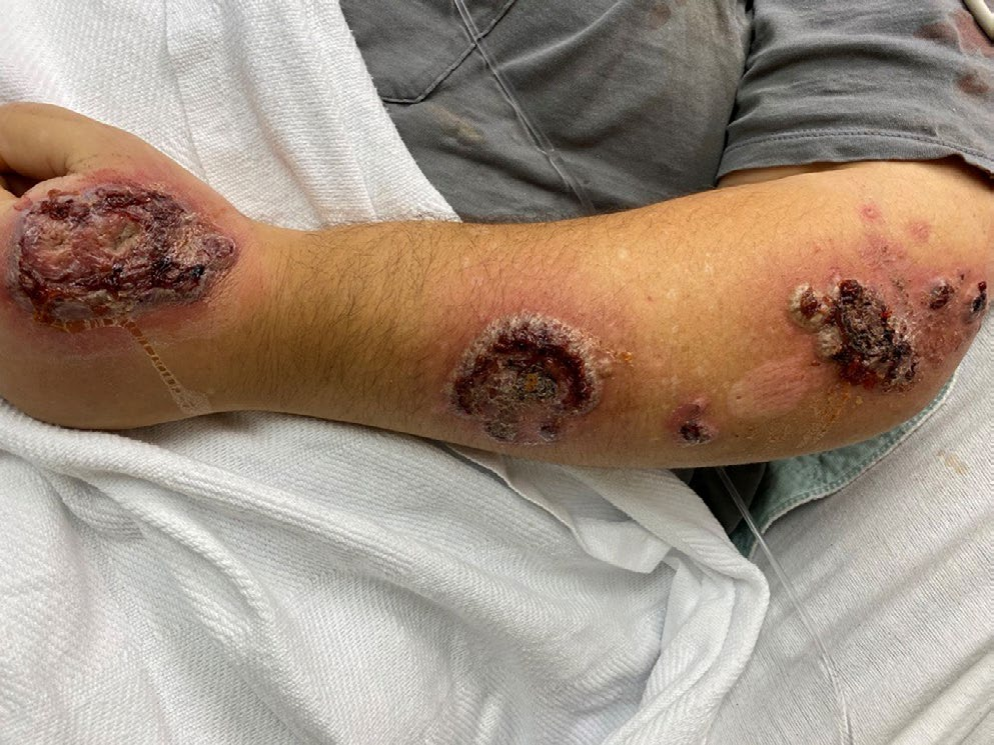
Multiple skin lesions of sweet syndrome

**Figure 2:**
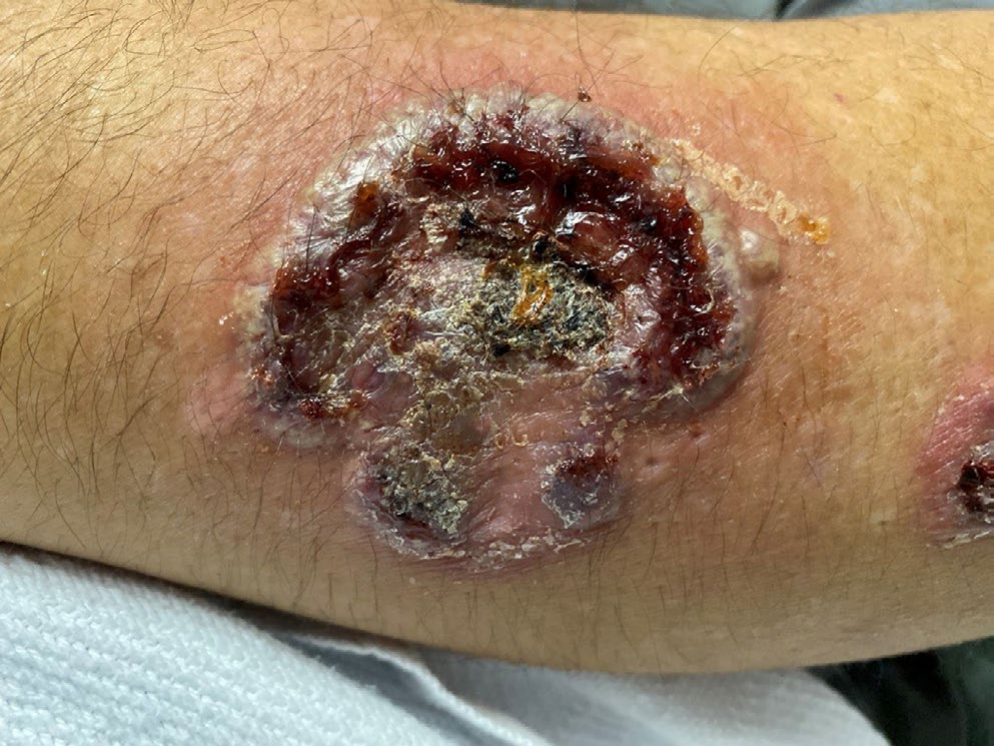
Close view of a single lesion of sweet syndrome

**Figure 3:**
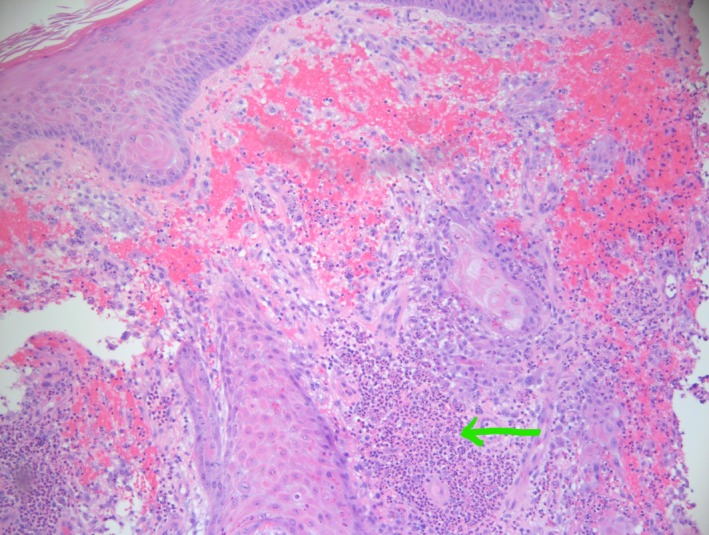
Hematoxylin and eosin staining showing intense neutrophil infiltrate (arrow pointing)

## CONFLICT OF INTEREST

None declared.

## AUTHOR CONTRIBUTION

LJ: took care of the patient in the hospital, helped in taking pictures, and writing manuscript; SM: took care of the patient in the hospital, edited manuscript, proof read the manuscript, did literature search, and added references.
